# Maternal blood parameters and risk of neonatal pathological jaundice: a retrospective study

**DOI:** 10.1038/s41598-023-28254-3

**Published:** 2023-02-14

**Authors:** Nan Jiang, Lu Qian, Guankai Lin, Yuxin Zhang, Sumiao Hong, Baochang Sun, Hexing Wang, Min Huang, Jiwei Wang, Qingwu Jiang

**Affiliations:** 1 Key Lab of Health Technology Assessment of Ministry of Health, School of Public Health, Fudan University, 130 Dong-An Road, Shanghai, 200032 China; 2 The People’s Hospital of Pingyang County, Wenzhou, 325400 Zhejiang China; 3 Wenzhou Center for Disease Control and Prevention, 490 Shifu Road, Wenzhou, 325000 Zhejiang China

**Keywords:** Risk factors, Paediatrics

## Abstract

This study aims to investigate the association between maternal blood parameters and the risk of neonatal pathological jaundice. A retrospective case–control study of 1309 newborns and their mothers from 2019 to 2020 in a single-center tertiary hospital. All mothers received a complete routine blood test prior to delivery, and outcome was neonatal pathological jaundice. We performed stepwise logistic regression modeling to identify maternal blood factors associated with neonatal pathological jaundice. 258 neonates (19.71%) were diagnosed with pathological jaundice. Logistic regression results showed that the odds ratio for pathological jaundice in neonates of mothers with high white blood cell (WBC) count was 1.512 (95% CI 1.145–1.998; *P* = 0.004). Besides, neonates whose mothers had a high mean corpuscular volume (MCV) during pregnancy doubled the odds of developing pathological jaundice (OR = 1.967; 95% CI 1.043–3.711; *P* = 0.037). Among neonates, those whose mothers had high levels of WBC count and MCV were at increased risk of pathological jaundice. Regular obstetric examinations and routine blood tests are essential to initiate adapted care.

## Introduction

Neonatal jaundice is one of the most common clinical conditions, occurring in approximately 80% of preterm infants and 60% of term infants during the first week of life^1,2^. It is often clinically manifested as a yellowish discoloration of the eye sclera and skin^3^. Neonatal jaundice can be divided into physiological jaundice and pathological jaundice. Physiological jaundice usually appears 2–3 days of life and resolves spontaneously without treatment^4^. Pathological jaundice, which accounts for about 25% of neonatal jaundice, is characterized by rapidly rising bilirubin concentrations in newborns^5,6^. It can cause bilirubin encephalopathy or kernicterus if not diagnosed and treated promptly^7^. Even survivors may suffer from various neurological sequelae such as hearing loss and gaze palsy^8^.

Neonatal pathological jaundice has been found to be influenced by a variety of medical, maternal and neonatal factors, such as gestational age, birth weight, family history of jaundice, maternal diseases and blood factors^9–13^. Routine blood test as a simple and inexpensive method is being recommended by more and more studies as an initial step to assist in the diagnosis of neonatal pathological jaundice^14,15^. Given the correlation between maternal and fetal hemograms^16^, abnormal maternal blood parameter levels may be important factors in reflecting the risk of neonatal jaundice early, but these links and mechanisms are poorly understood. Current research suggests a possible link between maternal blood factors and neonatal jaundice, such as maternal blood type may be a risk factor for neonatal jaundice^17^. In addition, white blood cell (WBC) count and the levels of hemoglobin (HGB) have also been found to be associated with an increased risk of neonatal jaundice^13,18,19^.

Moreover, there is convincing evidence that ethnic, seasonal and sociocultural factors influence the development of neonatal pathological jaundice^20–23^. For example, studies have found that jaundice in yellow babies is more severe and persistent than in white and black babies^24^. However, little research has been carried out in understanding the relationship between maternal blood factors and neonatal jaundice in China. In this context, it is crucial to first explore the relationship between maternal blood parameter levels and neonatal pathological jaundice.

This study aimed to investigate the effects of maternal blood parameter levels on neonatal pathological jaundice and to reduce the incidence and consequences of neonatal pathological jaundice by looking for modifiable risk factors.

## Materials and methods

### Study design and population

The data of this retrospective case–control study were collected from the Information System of The People's Hospital of Pingyang County. The economic development level of Pingyang County is in the middle level of Wenzhou city. In addition, The People's Hospital of Pingyang County is the only tertiary hospital responsible for the main medical and health services in Pingyang County. Most of the pregnant women in the area give birth in this hospital. However, it is not ruled out that a small number of pregnant women will choose to go to the tertiary hospital in the center of Wenzhou city, which will cause some bias. But in general, the data of this hospital is still representative of the overall situation of pregnant women in the region. Besides, this hospital has a good information system that records the demographic and clinical data of all patients. The case records of all mothers whose children had pathological jaundice hospitalized in the hospital from January 2019 to December 2021 were analyzed retrospectively. All controls were mothers from the same hospital at the same period, and none of their children developed pathological jaundice. All mothers in this study had singleton live births. Patients with incomplete information, defined as missing information on maternal blood parameters or neonatal health status, were excluded. This retrospective case–control study was approved by the Medical Research Ethics Committee of the School of Public Health, Fudan University (The international registry no. IRB00002408 and FWA00002399). Written informed consent was not required for this retrospective study because all patient identifying information was removed from the study data, requirement of consent was waived by the Medical Research Ethics Committee of the School of Public Health, Fudan University. All methods were performed in accordance with the relevant guidelines and regulations.

### Definition of neonatal pathological jaundice

The diagnostic criteria for neonatal pathological jaundice including (1) serum bilirubin level increases more than 85.0 μmol/L per day or more than 8.5 μmol/L per hour; (2) jaundice appears within 24 h after birth; (3) longer duration of jaundice, more than 2 weeks in full term infants and more than 4 weeks in preterm infants; (4) recurrent jaundice; (5) serum conjugated bilirubin level more than 34 μmol/L.

### Data collection

The following demographic and clinical data were collected: maternal age, neonatal sex, gestational diabetes mellitus (GDM), premature rupture of membranes (PROM), hyperemesis gravidarum, and the level of maternal blood parameters. Maternal blood parameters including WBC count (normal range: 3.5–9.5 × 10^9^/L), red blood cell count (RBC, normal range: 3.80–5.10 × 10^12^/L), HGB (normal range: 115–150 g/L), hematocrit (normal range: 0.35–0.45/L), mean corpuscular hemoglobin (normal range: 27.0–34.0 pg), mean corpuscular hemoglobin concentration (normal range: 316–354 g/L), mean corpuscular volume (MCV, normal range: 80.0–99.9 fL), platelet count (normal range: 100–300 × 10^9^/L), red cell distribution width—standard deviation (normal range: 37–54 fL), red cell distribution width—coefficient of variation (normal range:11.0–16.0 mmol/L), mean platelet volume (normal range: 7.8–11.0 fL), platelet distribution width (normal range: 7.0–17.1%), thrombocytocrit (normal range: 0.108–0.360/L), neutrophil ratio (normal range: 42.2–75.2%), absolute neutrophil count (ANC, normal range: 1.8–6.3 × 10^9^/L), absolute lymphocyte count (normal range: 1.1–3.2 × 10^9^/L), lymphocyte ratio (normal range: 20.5–51.1%), monocyte ratio (normal range: 3.0–10.0%), absolute monocyte count (normal range: 0.1–0.6 × 10^9^/L), absolute eosinophil count (normal range: 0.02–0.52 × 10^9^/L), eosinophil ratio (normal range: 0.4–5.0%), absolute basophil count (normal range: 0.0–0.1 × 10^9^/L), basophil ratio (normal range: 0.0–1.0%). Maternal blood parameter levels above the normal range were defined as high, and below the normal range as low.

### Statistical analysis

Data from Hospital Information System were managed using Microsoft Excel 365. Qualitative variables were presented as numbers and percentages. Chi-square test or Fisher’s exact test was used for inter-group comparison, where appropriate. For ranked variables, the Wilcoxon rank sum test was used to compare the differences between groups. To identify the independent risk factors for neonatal pathological jaundice, stepwise logistic regression analysis (bidirectional elimination, entry level of α = 0.05 and removal level of α = 0.10) was used. And we estimated the odds ratio (OR) and 95% confidence interval (95% CI). Statistical analysis was performed using the software STATA 15.1SE (STATA Corp, College Station, Texas, USA, http://www.stata.com) for Windows. Statistically significance was considered at a *p* value < 0.05.

### Ethical approval

This study was approved by the Medical Research Ethics Committee of the School of Public Health, Fudan University (The international registry Nos. IRB00002408 and FWA00002399).

## Results

Table 1 shows the demographics and clinical data of the 1309 neonates and their mothers. Mean maternal age was 29.76 ± 4.64 years old. Of 1309 singleton live births, 258 (19.71%) were diagnosed with neonatal pathological jaundice. About half of all newborns were male (51.18%). Among neonatal mothers, 113 (8.63%) of them were experiencing PROM, 39 (2.98%) of them had GDM during pregnancy, and 12 (0.92%) of them had hyperemesis gravidarum. The chance of neonatal pathological jaundice was statistically significantly different in PROM (*P* = 0.004), GDM (*P* = 0.030), WBC count (*P* = 0.003), and ANC (*P* = 0.012).

**Table 1:** Descriptive statistics and univariate analysis of maternal and neonatal with different characteristics.

Characteristics	N (%)	No pathological jaundice, N (%)	Pathological jaundice, N (%)	*P*
Number	1309	1051 (80.29%)	258 (19.71%)	
Age (years)				0.849^b^
18–34	1106 (84.49%)	889 (80.38%)	217 (19.62%)	
35–46	203 (15.51%)	162 (79.80%)	41 (20.20%)	
Neonatal sex				0.051^b^
Male	670 (51.18%)	552 (82.39%)	118 (17.41%)	
Female	639 (48.82%)	499 (78.09%)	140 (21.91%)	
Premature rupture of membranes				0.004^b^
No	1196 (91.37%)	972 (81.27%)	224 (18.73%)	
Yes	113 (8.63%)	79 (69.91%)	34 (30.09%)	
Gestational diabetes mellitus				0.030^b^
No	1270 (97.02%)	1025 (80.71%)	245 (19.29%)	
Yes	39 (2.98%)	26 (66.67%)	13 (33.33%)	
Hyperemesis gravidarum				0.139^a^
No	1297 (99.08%)	1039 (80.11%)	258 (19.89%)	
Yes	12 (0.92%)	12 (100%)	0 (0%)	
Red cell distribution width—coefficient of variation				0.303^a^
Normal	1265 (96.64%)	1013 (80.08%)	252 (19.92%)	
High	44 (3.36%)	38 (86.36%)	6 (13.64%)	
Red cell distribution width—standard deviation				0.154^c^
Low	28 (2.14%)	24 (85.71%)	4 (14.29%)	
Normal	1265 (96.64%)	1012 (80.00%)	253 (20.00%)	
High	16 (1.22%)	15 (93.75%)	1 (6.25%)	
Monocyte ratio				0.922^c^
Low	5 (0.38%)	4 (80.00%)	1 (20.00%)	
Normal	1293 (98.78%)	1038 (80.28%)	255 (19.72%)	
High	11 (0.84%)	9 (81.82%)	2 (18.18%)	
Absolute monocyte count				0.116^b^
Normal	1036 (79.14%)	841 (81.18%)	195 (18.82%)	
High	273 (20.86%)	210 (76.42%)	63 (23.58%)	
Lymphocyte ratio				0.816^b^
Low	885 (67.61%)	709 (80.11%)	176 (19.89%)	
Normal	424 (32.39%)	342 (80.66%)	82 (19.34%)	
Absolute lymphocyte count				0.784^c^
Low	45 (3.44%)	36 (80.00%)	9 (20.00%)	
Normal	1257 (96.03%)	1010 (80.35%)	247 (19.65%)	
High	7 (0.53%)	5 (71.43%)	2 (28.57%)	
White blood cell count				0.003^b^
Normal	720 (55.00%)	599 (83.19%)	121 (16.81%)	
High	589 (45.00%)	452 (76.74%)	137 (23.26%)	
Red blood cell count				0.402^c^
Low	479 (36.59%)	390 (81.42%)	89 (18.58%)	
Normal	818 (62.49%)	651 (79.58%)	167 (20.42%)	
High	12 (0.92%)	10 (83.33%)	2 (16.67%)	
Hematocrit				0.269^c^
Low	515 (39.34%)	423 (82.14%)	92 (17.86%)	
Normal	790 (60.35%)	626 (79.24%)	164 (20.76%)	
High	4 (0.31%)	2 (50.00%)	2 (50.00%)	
Mean corpuscular volume				0.805^c^
Low	75 (5.73%)	65 (86.67%)	10 (13.33%)	
Normal	1185 (90.53%)	952 (80.34%)	233 (19.66%)	
High	49 (3.74%)	34 (69.39%)	15 (30.61%)	
Platelet count				0.340^c^
Low	4 (0.31%)	4 (100%)	0 (0%)	
Normal	1229 (93.89%)	990 (80.55%)	239 (19.45%)	
High	76 (5.81%)	57 (75.00%)	19 (25.00%)	
Mean platelet volume				0.464^c^
Low	46 (3.51%)	39 (84.78%)	7 (15.22%)	
Normal	1122 (85.71%)	897 (79.95%)	225 (20.05%)	
High	141 (10.77%)	115 (81.56%)	26 (18.44%)	
Platelet distribution width				0.826^a^
Normal	1276 (97.48%)	1025 (80.33%)	251 (19.67%)	
High	33 (2.52%)	26 (78.79%)	7 (21.88%)	
Thrombocytocrit				0.201^c^
Low	9 (0.69%)	8 (88.89%)	1 (11.11%)	
Normal	1294 (98.85%)	1037 (80.14%)	257 (19.86%)	
High	6 (0.46%)	6 (100%)	0 (0%)	
Neutrophil ratio				0.684^b^
Normal	720 (55.00%)	581 (80.69%)	139 (19.31%)	
High	589 (45.00%)	470 (79.80%)	119 (20.20%)	
Absolute neutrophil count				0.012^b^
Normal	505 (38.58%)	423 (83.76%)	82 (16.24%)	
High	804 (61.42%)	628 (78.11%)	176 (21.89%)	
Absolute basophil count				
Normal	1309	1051 (80.29%)	258 (19.71%)	
Basophil ratio				
Normal	1309	1051 (80.29%)	258 (19.71%)	
Eosinophil ratio				0.139^c^
Low	177 (13.52%)	137 (77.40%)	40 (22.60%)	
Normal	1127 (86.10%)	912 (80.92%)	215 (19.08%)	
High	5 (0.38%)	2 (40.00%)	3 (60.00%)	
Absolute eosinophil count				0.180^c^
Low	422 (32.24%)	334 (79.15%)	88 (20.85%)	
Normal	883 (67.46%)	717 (81.20%)	166 (18.80%)	
High	4 (0.31%)	0 (0%)	4 (100%)	
Mean corpuscular hemoglobin concentration				0.119^b^
Low	119 (7.53%)	102 (85.71%)	17 (14.29%)	
Normal	1189 (92.47%)	949 (79.30%)	241 (20.70%)	
Mean corpuscular hemoglobin				0.287^c^
Low	156 (11.92%)	133 (85.26%)	23 (14.74%)	
Normal	1139 (87.01%)	909 (79.81%)	230 (20.19%)	
High	14 (1.07%)	9 (64.29%)	5 (35.71%)	
Hemoglobin				0.097^c^
Low	536 (40.95%)	443 (82.65%)	93 (17.35%)	
Normal	771 (58.90%)	607 (78.73%)	164 (21.27%)	
High	2 (0.15%)	1 (50.00%)	1 (50.00%)	

^a^*P* value was derived from the Fisher’s exact test.

^b^*P* value was derived from the chi-square test.

^c^*P* value was derived from the Wilcoxon rank sum test.

A stepwise logistic regression model was constructed to investigate the effects of maternal age, maternal diseases, maternal blood parameters and neonatal sex on the occurrence of neonatal pathological jaundice. Maternal with PROM (OR = 1.965; 95% CI 1.271–3.036; *P* = 0.002), GDM (OR = 2.067; 95% CI 1.038–4.118; *P* = 0.039), High MCV (OR = 1.967; 95% CI 1.043–3.711; *P* = 0.037) and high WBC count (OR = 1.512; 95% CI 1.145–1.998; *P* = 0.004) were found to be independent risk factors for neonatal pathological jaundice (Table 2).

**Table 2:** Stepwise logistic regression analysis on independent risk factors for neonatal pathological jaundice.

Variables	OR	*P*	95% CI
PROM			
No	Reference		
Yes	1.965	0.002	(1.271, 3.036)
GDM			
No	Reference		
Yes	2.067	0.039	(1.038, 4.118)
MCV			
Normal	Reference		
High	1.967	0.037	(1.043, 3.711)
WBC count			
Normal	Reference		
High	1.512	0.004	(1.145, 1.998)
Constant	0.179		

## Discussion

We conducted a retrospective case–control study to comprehensively investigate the relationship between maternal blood parameter levels and neonatal pathological jaundice. Among neonates, those whose mothers had high levels of WBC count and MCV were at increased risk of pathological jaundice. The associations provide clues for further exploration of physiopathological mechanisms.

19.71% of neonates in this study were diagnosed with pathological jaundice. This result was higher than the finding from Ethiopia (5.99%)^6^, Nepal (9.20%)^25^ and Romania (0.49%)^26^, but it is close to the previous Chinese study (14.3%)^24^. This increased incidence of neonatal pathological jaundice could be due to the regional and sociocultural differences in the study population. For example, studies have shown that the use of some herbs by Chinese pregnant women may cause jaundice in newborns^20^. Additionally, neonatal jaundice is more severe in yellow races than other races^24^. Differences in study time and design could also contribute to this inconsistency in incidence^3^. Besides, this study was consistent with other studies indicating that the development of neonatal pathological jaundice was not related to neonatal sex^27^. There was also no significant correlation between maternal age and neonatal pathological jaundice in this study. The results of a study from Taiwan were consistent with our study^28^. However, studies from the United States^29^, Tehran^13^ and other regions^10^ have found a significant association between maternal age and neonatal jaundice. Given the geographic proximity of Taiwan and Zhejiang, this may indicate regional differences in risk factors for neonatal pathological jaundice, and further multi-regional studies are needed to explore this difference.

This study revealed that the odds of developing pathological jaundice among neonates whose mothers had PROM or GDM were almost twice higher compared with those neonates whose mothers without these conditions. This finding was supported by existing research evidence. Mothers with PROM were at increased risk of preterm birth, which may be the major cause of pathological jaundice in their newborns^30^. In addition, neonates of mothers with GDM may be deficient in energy provision due to phosphorylation disorder, resulting in elevated serum bilirubin levels^31^.

It was also found maternal with higher-than-normal WBC counts were 51.2% (95% CI 14.5–99.8%) more likely to develop pathological jaundice in their newborns. This is consistent with existing studies that found a significant correlation between maternal WBC count and neonatal jaundice^13^. WBC count is a typical clinical marker for bacterial infection, and a high level of maternal WBC count indicates that the mother may be at risk of bacterial infection^32^. This could further lead to bacterial infection of the newborn, causing premature destruction of the newborn's RBC, resulting in pathological jaundice^4^.

Moreover, this study suggested that increased levels of MCV had a significant effect on neonatal pathological jaundice. Maternal with higher-than-normal MCV were 96.7% (95% CI 4.3–271.1%) more likely to develop pathological jaundice in their newborns. MCV is a value that describes the average size and volume of red blood cells in a blood sample^33^. An increase in MCV in pregnant women suggested possible folate or vitamin B12 deficiency^34^. During pregnancy, folate and vitamin B12 could be transferred from the mother to the fetus through the placenta^35^. Therefore, maternal folate or vitamin B12 deficiency could lead to folate and vitamin B12 deficiency in the newborn. In this condition, the RBC cannot mature adequately results in hemolysis and pathological jaundice in neonates^36,37^. However, a cross-sectional study from Tehran showed that maternal MCV did not increase the risk of neonatal jaundice^13^. In addition, a population-based cohort study of more than one million newborns found that maternal blood factors were the dominant risk factors for haemolytic jaundice, while nonhaemolytic jaundice was mainly affected by pregnancy factors^17^. Therefore, further studies are needed to elucidate the role of maternal blood factors, such as MCV, in various types of neonatal pathological jaundice.

This study has some limitations. First, some potential risk factors were not included in the study because information such as socioeconomic factors and body mass index of pregnant women were not available in the hospital information system. Furthermore, the data for this study came from a single center, which was underrepresented relative to the huge Chinese population. However, the results of this study are still of great significance for reducing the incidence and mortality of neonatal pathological jaundice, especially in developing countries.

## Conclusions

The findings highlight the potential link between maternal blood parameter levels and neonatal pathological jaundice and suggest that future studies should investigate the mechanisms by which WBC count and MCV affect the pathogenesis of neonatal jaundice. Therefore, pregnant women were recommended to have regular obstetric examinations and routine blood tests during pregnancy, paying particular attention to changes in WBC count and MCV. The incidence of neonatal pathological jaundice in The People's Hospital of Pingyang County was high. Early intervention is warranted for pregnant women at high risk for neonatal pathological jaundice, especially in the study area.

## Data Availability

The datasets used to support the findings of this study are available from the corresponding author on reasonable request.

## Abbreviations

OR: Odds ratio
CI: Confidence interval
PROM: Premature rupture of membranes
GDM: Gestational diabetes mellitus
WBC: White blood cell
RBC: Red blood cell
HGB: Hemoglobin
MCV: Mean corpuscular volume
ANC: Absolute neutrophil count
